# Complete Genome Sequence of Stenotrophomonas maltophilia AB550, an Environmental Solar Radiation- and Multidrug-Resistant Strain Isolated in Western Australia

**DOI:** 10.1128/MRA.00914-18

**Published:** 2018-08-09

**Authors:** Ratish R. Permala, Julie Glady-Croue, Elizabeth L. J. Watkin, Joshua P. Ramsay, Jean-Philippe Croue

**Affiliations:** a School of Pharmacy and Biomedical Sciences, Curtin University, Perth, Western Australia, Australia; b Curtin Health Innovation Research Institute, Curtin University, Perth, Western Australia, Australia; c Curtin Water Quality Research Centre, Curtin University, Perth, Western Australia, Australia; Indiana University Bloomington

## Abstract

We report here the complete genome sequence of Stenotrophomonas maltophilia AB550, a multidrug- and solar radiation-resistant strain isolated from the effluents of an urban wastewater treatment plant in Western Australia. The genome consists of a single 4.9-Mb chromosome.

## ANNOUNCEMENT

Stenotrophomonas maltophilia is frequently isolated from aqueous environmental sources, in both medical and nonclinical settings, and is often resistant to multiple classes of antibiotics. The increasing incidence of hospital-acquired S. maltophilia infection highlights the serious threat to human health posed by these drug- and treatment-resistant isolates (1–3).

S. maltophilia strain AB550 was isolated from a wastewater treatment plant in Western Australia following treatment with artificial solar radiation. AB550 is resistant to multiple antibiotics, and previous draft genome sequencing indicated that it carries genes coding for predicted efflux pumps, beta-lactamases, and aminoglycoside-modifying enzymes (4).

Total AB550 genomic DNA was extracted from lysogeny broth (LB) culture using a genomic DNA minikit (Favorgen). Whole-genome sequencing was performed with Paciﬁc Biosciences (PacBio) RS II sequencing technology using one single-molecule real-time (SMRT) cell (Macrogen, South Korea). PacBio sequencing generated 142,190 postﬁlter subreads with an average length of 10,654 bp. Genome assembly was performed using Canu (v. 1.6), and annotation was performed using the NCBI Prokaryotic Genome Annotation Pipeline (https://www.ncbi.nlm.nih.gov/genomes/static/Pipeline.html). The Canu assembly output indicated that the single generated contig was likely circular. The average depth of coverage was 306-fold, and the G+C content of the genome was 66.5%. The genome was assembled into a single circular contig of 4,943,426 bp. A total of 4,611 genes were identified, of which 4,392 were coding sequences (CDS), 94 were RNA genes, and 125 were apparent pseudogenes.

### Data availability.

The complete genome sequence of AB550 has been deposited in GenBank under the accession number CP028899.
